# Safety Evaluation of *Bifidobacterium lactis* BL-99 and *Lacticaseibacillus paracasei* K56 and ET-22 *in vitro* and *in vivo*

**DOI:** 10.3389/fmicb.2021.686541

**Published:** 2021-07-29

**Authors:** Hongyun Lu, Wen Zhao, Wei-Hsien Liu, Ting Sun, Hanghang Lou, Tianyu Wei, Wei-Lian Hung, Qihe Chen

**Affiliations:** ^1^Department of Food Science and Nutrition, Zhejiang University, Hangzhou, China; ^2^Inner Mongolia Dairy Technology Research Institute Co., Ltd., Hohhot, China; ^3^Yili Innovation Center, Inner Mongolia Yili Industrial Group Co., Ltd., Hohhot, China

**Keywords:** probiotics, *Bifidobacterium lactis*, safety assessment, toxicity, *Lacticaseibacillus paracasei*

## Abstract

Probiotics have been reported to play a major role in maintaining the balance of microbiota in host. Consumption of food with probiotics has increased with consumer concerns regarding healthy diets and wellness. Correspondingly, safety evaluation of probiotics for human consumption has become increasingly important in food industry. Herein, we aimed to test the safety of *Bifidobacterium lactis* BL-99 and *Lacticaseibacillus paracasei* K56 and ET-22 strains *in vitro* and *in vivo*. In results, these strains were found to be negative for mucin degradation and platelet aggregation test. Additionally, the three strains were susceptible to eight antibiotics. In accordance with bacterial reversion mutation (Ames) assay, the tested strains had no genetic mutagenicity. Finally, it was confirmed that there were no dose-dependent mortality and toxicity throughout multidose oral toxicity tests in rats. Our findings demonstrated that *B. lactis* BL-99 and *L. paracasei* K56 and ET-22 can achieve the generally recognized as safe (GRAS) status as probiotics in the future.

## Introduction

Probiotics are defined as “live microorganisms which when administered in adequate amounts confer a health benefit on the host” (Hill et al., 2014). It was suggested that a daily intake of 10^8^–10^9^ CFU/g probiotic bacteria could survive the upper ingestion to exert their positive physiological functions in the human body (Knorr, 1998; Dimitrellou et al., 2019). Recently, the global market for probiotics has been increasingly growing guided by the rising consumers’ demand for healthy diets and wellness, which caused food researchers and industries to develop new probiotic-containing products and study specific characteristics of probiotics. Among the different microbial types, several *Bifidobacterium* and *Lactobacillus* species of probiotics have been developed and marketed. Probiotics, mainly *Lactococcus*, *Lactobacillus*, and *Bifidobacterium* commercially used in food industry are live microorganisms that inhabited the gut and possess versatile health-promoting properties, modulating the human gastrointestinal microbiota through inhibiting the growth of opportunistic bacteria (Jessie Lau and Chye, 2018; Zheng et al., 2020). At present, *bifidobacteria* and *L. casei* with good physiological activity increasingly have been discovered as probiotics. It has been revealed by genome mining that probiotic *Lactobacillus* and *Bifidobacterium* strains possessed safety characteristics, antiviral activities, and host adherence factors (Abdelhamid et al., 2019). *Bifidobacteria*, members of the human gut microbiota, have shown to exert health-promoting effects on their hosts, such as reducing of diarrhea, establishment of a healthy microbiota, modulation of immune systems, improvement of lactose intolerance, cholesterol reduction, and cancer prevention (O’Callaghan and van Sinderen, 2016; Alessandri et al., 2019). *L. casei*, a *Lacticaseibacillus* species which contains probiotic strains, has been demonstrated to have many health-promoting benefits (Liu et al., 2011; Ivanovska et al., 2017; Sperry et al., 2018). Among them, certain strains have a long history of safe and effective use as probiotics. With regard to safety, most species of the *Lactobacillus* genus have been safely administered to newborn infants, immunocompromised, and critically ill patients without apparent adverse effects (Ladas et al., 2016), regarded as “Generally Recognized As Safe”(GRAS) (Salminen et al., 1998). However, the safety of potential probiotics should not be overlooked. The probiotic effects are strain specific (Espitia et al., 2016). Introducing a new probiotic strain demands that it is at least as safe as its conventional counterparts. Hence, safety assessment is a must before probiotics are used for human applications. There were ample documented evidences of safe use of *Lactobacillus* and *Bifidobacterium*; few cases of bacteraemia and endocarditis have also been reported among patients with severe diseases such as short gut syndrome, heart valve transplantation, and severe, active ulcerative colitis albeit at a low frequency (Meini et al., 2015; Harding-Theobald and Maraj, 2018; Meleh et al., 2020). Therefore, the safety of probiotics is of paramount importance as new strains are continuously evolving and being commercialized. Currently, there are diverse generally accepted method to evaluate the safety of probiotics. The safety evaluation methods of probiotics mainly focus on *in vitro* experiments including platelet aggregation ability, antibiotic susceptibility, mucin degradation, bacterial recurrent mutation, and *in vivo* oral toxicity test (Pradhan et al., 2020).

To this end, the safety aspect of *B. lactis* BL-99 and *L. paracasei* K56 and ET-22 strains were tested according to the Joint FAO/WHO guidelines (FAO/WHO, 2020). Firstly, *in vitro* tests were performed to analyze mucin degradation and platelet aggregation activities, to investigate whether these strains have the potential to disrupt the intestinal barrier and trigger platelet aggregation to induce diseases. Secondly, Ames study were conducted to determine whether they have mutagenicity. Finally, multidose oral toxicity tests were performed in rats to assess the safety of the aforesaid strains.

## Materials and Methods

### Bacterial Strains and Growth Conditions

Unadulterated cultures of *Bifidobacterium animalis* subsp. *lactis.* BL-99 (CGMCC No.15650) and *L. paracasei* K56 (CGMCC 15139 and DSM27447) and ET-22 (CGMCC No. 15077) were isolated from the intestines of 2-year-old Chinese healthy infant (Shanghai, China) and have been identified by 16S rRNA gene. For genotype identification, 16S rRNA gene of the BL-99 strains were amplified using the universal primers 27F (5′-AGA GTT TGA TCC TGG CTC AG-3′) and 1492R (5′-GGT TAC CTT GTT ACG ACT T T-3′) (Li et al., 2010). The primer of K56 and ET-22 16S rRNA gene were designed based on this site and called LbLMA1-rev (5′-CTC AAA ACT AAA CAA AGT TTC-3′). A second primer R16-1 (5′-CTT GTA CAC ACC GCC CGT CA-3′) (Pepper and Britz, 2019). The standard bacterial culture was proliferated with MRS medium (Solarbio, Beijing) and stored in 40% glycerol at −80°C for further use. *Bifidobacteria* grow anaerobically. Anaerobic environment was obtained with Anaero Gen sachets (Oxoid Ltd., West Heidelberg/VIC, Australia).

*B. lactis* BL-99 and *L. paracasei* K56 and ET-22 further were manufactured under 21 CFR 111, Current Good Manufacturing Practice (GMP) in Manufacturing, Packaging, Labeling, or Holding Operations for Dietary Supplements (US FDA, 2015). The proprietary manufacturing process was a batch-type fermentation using sterilized media comprising proteins, carbohydrates, vitamins, and minerals in water prior to inoculation with the selected bacteria. Each batch of each strain was fermented and freeze-dried individually and required to pass quality checks for enumeration, identity. The product was always formulated to contain viable cells at or above the 1.5 × 10^11^ CFU/g same as the label claim until the labeled expiration date at recommended storage conditions in −20°C.

### *In vitro* Toxicity Study

#### Mucin Degradation Test

According to previous reports (Abe et al., 2010; Kim et al., 2018), mucin degradation tests were performed with slight modifications. Briefly, three strains of *L. paracasei* K56 and ET-22 and *B. lactis* BL-99 were inoculated into 10 ml sugar-free MRS basal medium with or without 0.3% purified mucin (Sigma-Aldrich, St. Louis, MO, United States) and 1% glucose, cultured at 37°C aerobic conditions for 48 h. The sugar-free MRS basal medium consisted of 10 g/L peptone, 5 g/L beef extract powder, 4 g/L yeast extract powder, 5 g/L sodium acetate, 2 g/L triammonium citrate, 2 g/L K_2_HPO_4_, 1 g/L Tween 80, 0.2 g/L MgSO_4_, and 0.05 g/L MnSO_4_. After incubation for 12, 24, 36, and 48 h, bacterial growth was evaluated by the microplate photometer at 600 nm (MultiSkan FC, Thermo Fisher Scientific K.K., Tokyo, Japan) and pH of the culture. To further confirm mucin degradation, SDS-PAGE was also used for the research (Zhou et al., 2001).

#### Platelet Aggregation Test

The healthy rabbits weighing about 2–3 kg were injected with 1% pentobarbital sodium solution at the auricular border at a dose of 3 ml/kg. The rabbits were completely anesthetized, and blood was collected by intubation in both tracheal arteries; 3.2% sodium citrate solution was used for anticoagulation at a ratio of 1:9 (anticoagulant:blood). The whole blood was centrifuged at 93 × *g* for 12 min, and the supernatant was taken to platelet-rich plasma (PRP). The rest of the blood was centrifuged at 2,325 × *g* for 12 min, and the supernatant was platelet-poor plasma (PPP). PPP was used to adjust the concentration of PRP to 3 × 10^8^/ml. Four hundred microliters of PPP was taken for the base test, and the light transmittance of the platelet aggregator was automatically set to 0%. Then, 400 μl PRP was taken into a colorimetric cup, 50 μl of 10 μg/ml agonist arachidonic acid (Sigma-Aldrich, St Louis, MO, United States), and 50 μl tested strain (10^7^ CFU/ml) or negative control (PBS) were added and incubated at 37°C for 5 min, then the tested tubes were placed in the room temperature for reaction. The platelet aggregation rate was tested after stirring within 5 min. The platelet aggregation rate of tested samples can be calculated by the following formula:

Aggregation rate =(maximum aggregation rate of test group−maximum aggregation rate of blank group)/(maximum aggregation rate of blank group)×100%

#### Bacterial Reversion Mutation Assay (Ames Test)

According to Zhang et al. (2021), the plate infiltration method was used to test the Ames test using the non-metabolic activation system (S9) of the tested strain. Five dose groups of tested strains were set at five-time intervals, namely, 5,000, 1,000, 200, 40, and 8 μg/dish. Additionally, spontaneous regression group, solvent control, and positive control were also set and 5 g samples were dissolved in 100 ml sterile distilled water, and then the samples were inactivated after boiling water bath for 30 min. After 5%, mother liquor was prepared as the highest dose, sterile distilled water was used to dilute the mother liquor five times in turn to the required dose of the tested solution, and the solution was shaken well for the test. The experiment was carried out under the condition of adding S9 and without S9. After being cultured at 37°C for 48 h, the number of colonies per dish in each tested group was recorded. Ames test was determined to be positive when the number of regressive colonies in the subject group was more than doubled (the number of regressive colonies is equal to or greater than two times the number of solvent controls) and there was a dose-response relationship or at least a reproducible and statistically significant positive reaction at one of the tested sites.

#### Antibiotic Susceptibility

The antibiotic susceptibility of the tested strains was assessed on MRS agar plates using the antibiotic disc diffusion method and measurement of minimum inhibition concentration (MIC). Referred to references with minor revision (Wu et al., 2016; Somashekaraiah et al., 2019), 1 ml of tested strains (about 1.5 × 10^8^ CFU/ml) was absorbed on 15 ml MRS medium, respectively, and mixed by a whirlpool oscillator and poured into a petri dish. After the medium solidified, the drug-sensitive paper was attached to the medium surface with sterile forceps. Then, the tested strains were placed in an incubator at 37°C for upside-down culture. After cultured for 48 h, the diameter of the inhibition zone was measured and recorded with a vernier caliper with an accuracy of 0.02 mm. The resistance of the tested strains to eight antibiotics was according to the American Association for Clinical and Laboratory Standardization (CLSI, 2015). The results were subjected to a qualitative classification of microorganisms as sensitive, moderately susceptible, or resistant to the antimicrobial drug tested.

According to the microbiological cut-off value of bacteria in animal feed additives prescribed by the European Food Safety Authority (EFSA) in 2012 guidelines (EFSA, 2012), measurement of MIC was performed as follows. The strains BL-99, K56, and ET-22 were cultured in MRS liquid medium at 37°C for 24–48 h, then the bacteria liquids were coated or streamed onto MRS plate for 48 h at 37°C. Single or multiple bacterial colonies were selected from the plate and resuspend into 5 ml 0.85–0.9% normal saline, and their OD_600_ was adjusted to 0.2. Take 250 μl resuspend for 100-fold dilution and add to 25 ml LSM broth (Huys et al., 2010). One hundred microliters of diluent was inoculated into 96-well plates containing antibiotic medium with different dilution concentrations, each dilution concentration was repeated for three times, and the final concentration of the bacteria was 10^5^ CFU/ml. After being cultured at 37°C for 24 h, the OD_600_ value of bacteria in each well was determined. MIC values were read as the lowest concentration of an antibiotic agent at which visible growth was inhibited, in comparison with an antibiotic-free control well. Strains showing MIC values less than the breakpoints of EFSA were considered sensitive. Otherwise, they were recorded to be resistant.

### *In vivo* Toxicity Study

#### Animals and Tested Organisms

ICR mice weighing 18–22 g and Sprague Dawley (SD) rats (SPF grade) weighing 50–100 g were provided by Zhejiang Provincial Laboratory Animal Center. All the *in vivo* studies were conducted as per current legislation on animal experiments with license No. SYXK (Zhejiang) 2014-0008. Animal experiments were completed in barrier system laboratory (air cleanliness ≤ 10,000 level, air ventilation frequency 10–20 times/h, temperature 20–26°C, daily temperature difference ≤ 3°C, relative humidity 40–70%). The rats were fed with ultrafiltration water and nutritive compound feed sterilized by 60 co-irradiation. Additionally, cages and bedding materials were autoclaved at 121°C and sterilized for 20 min. The mice were randomly divided into four groups fed with phosphate-buffered saline (PBS), ET-22, K56, and BL-99, respectively. The animals were then acclimatized for 5 days before the experiment.

#### Acute Oral Toxicity Study (14 Days Repeated Dose)

At the end of adaptive feeding, male and female mice were randomly selected and divided into four groups with 10 males and 10 females in each group, respectively. Oral gavage was given once a day at a dose of 3 × 10^12^ CFU/kg BW for 14 days. The behavior, death, and poisoning of the mice were observed and recorded after inoculation with bacterial suspension. Moreover, average weight per mouse was calculated. After the test, mice were killed, and the heart, liver, spleen, lung, and kidney were recorded.

#### Subacute Oral Toxicity Study (28 Days Repeated Oral Dose)

After adaptive feeding, half male and half female rats were randomly selected and divided into 10 groups with 20 rats in each group. Three concentration gradients of high (H), medium (M = H/2) and low (L = H/4) were set for the inoculation of bacterial powder. Among them, the maximum intragastrical doses (H dose) of K56, ET-22, and BL-99 were 5.25 × 10^11^, 5.25 × 10^11^, and 7.5 × 10^11^ CFU/kg, respectively. The rats in the control group were given the same amount of sterilized PBS and administered intragastrically once a day for 28 days. The behavior, death, and poisoning signs of the rats were observed and recorded. At the end of the experiment, the weight gain and total food intake were calculated.

#### Hematology and Blood Biochemistry

After the subacute toxicity test, blood samples were taken from the rats. The recommended indicators for hematology are white blood cell (WBC) count, red blood cell (RBC) count, hemoglobin concentration, hematocrit, platelet count, etc. Indicators of blood biochemistry include electrolyte balance, glucose, lipid and protein metabolism, liver (cell, bile duct) renal function, etc., including alanine aminotransferase, aspartate aminotransferase, glutamyl transpeptidase, alkaline phosphatase, urea, creatinine, blood glucose, total protein, albumin, total cholesterol, triglyceride, chlorine, potassium, and sodium indicators.

#### Histopathological Examination

At the end of the subacute toxicity test, the highest dose group and the control group of rats in the biological safety cabinet viscera histopathological examination, inspection organs shall include the following: brain, thyroid, thymus, heart, liver, spleen, kidney, adrenal gland, stomach, duodenal, colon and mesenteric lymph nodes, ovaries, testes, and bladder. The organs were fixed with 4% paraformaldehyde, and pathological tissue sections were made for analysis. The fixed tissue segments were embedded in paraffin and stained with H&E (Sigma-Aldrich, St. Louis, MO, United States) for histological assessment under a light microscope.

#### Bacterial Translocation

The bacterial translocation test was referred to Umar et al. (2020), with minor modification. Rats fed for 28 days were killed and dissected in a sterile environment. Heart, liver, spleen, lung, kidney, and other viscera were taken with a sterile scalpel and cut open. One gram of the respective tissue samples was homogenized in sterile PBS, and 100 μl of the homogenates was spread onto MRS and brain heart infusion (BHI) agar. The organs were then anaerobically cultured at 37°C for 24–48 h to observe whether there was a characteristic bacterial colony growth. In case of presence of any microbe in the tissue samples, randomly amplified polymorphic DNA (RAPD) finger-printing method (Pradhan et al., 2019) would be used to identify the translocated microorganisms in tissue samples with the help of selected primers with arbitrary nucleotide sequences (5′-AACTGGCCCC-3′ and 5′-CCGGGCAAGC-3′).

### Statistical Analysis

All the data obtained in this study were presented as mean ± SD and analyzed statistically with the Origin 9.0 software. Statistical significance among different groups was compared by one-way ANOVA following Tukey’s *post-hoc* test and considered significant at *p* ≤ 0.05.

## Results

### Mucin Degradation Test

**FIGURE 1:**
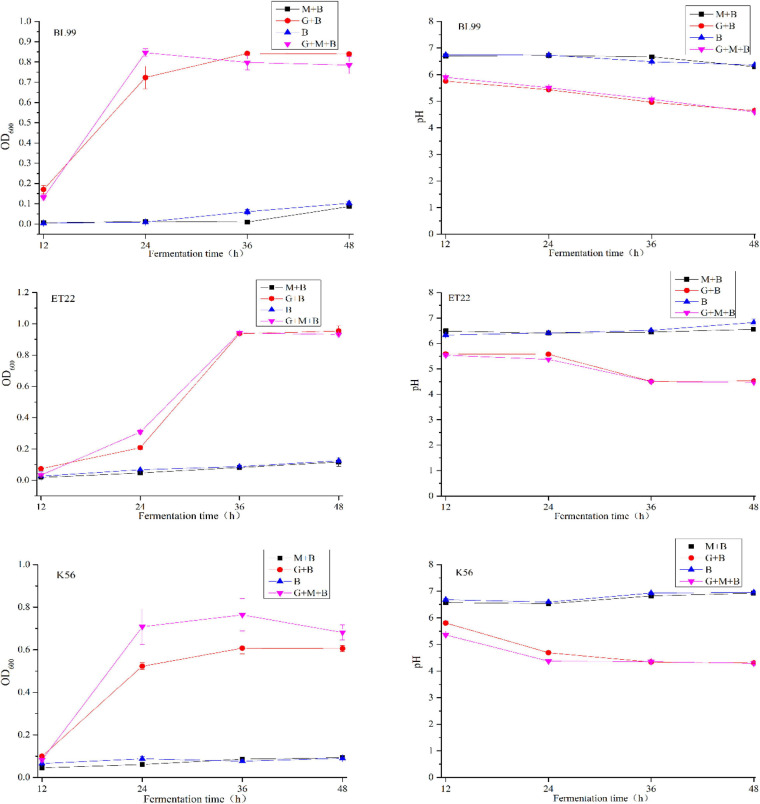
The OD_600_ and pH values of BL-99, ET-22, and K56 strains were measured within 12, 24, 36, and 48 h of fermentation culture. The four different media are basal medium (B), basal medium containing 1% glucose (G+B), basal medium containing 0.3% gastrointestinal mucosal protein (M+B), basal medium containing 1% glucose, and 0.3% gastrointestinal mucosal protein (G+M+B).

**FIGURE 2:**
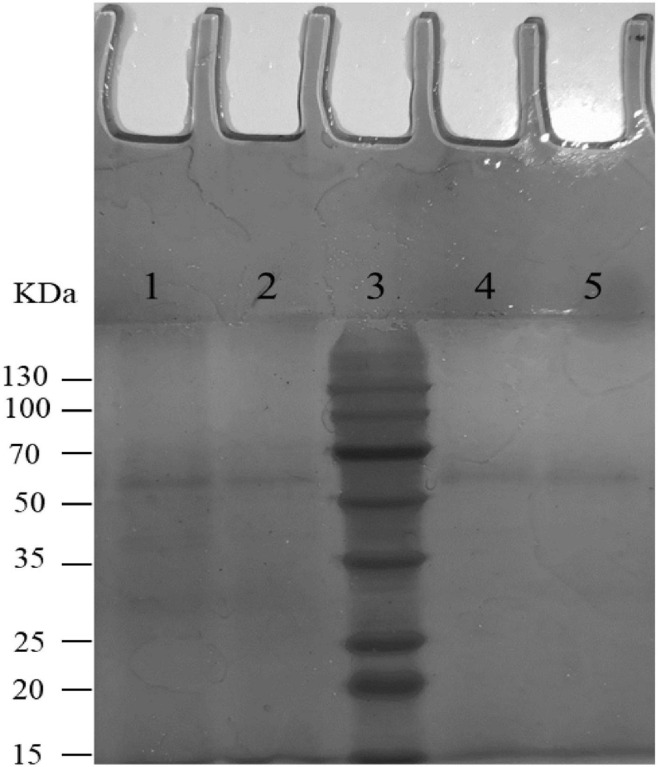
SDS-PAGE analysis of mucin degradation by three tested strains. Lanes 1–5 were negative controls (uncultured mucin fluid), BL-99, marker, ET-22, and K56, respectively.

The degradability of BL-99, ET-22, and K56 to gastrointestinal mucosa was determined by different modified media. Cell growth rate was measured by measuring the optical density at 600 nm (OD_600_) and the pH of the culture. According to Figure 1, the OD_600_ value of ET-22, BL-99, and K56 in the carbon-free basal medium (B) and 0.3% gastrointestinal mucosa protein basal medium (M+B) was significantly lower than that in the medium containing 1% glucose and was close to 0. Moreover, when the three tested strains only used mucin as carbon source, the pH of the culture medium would not be significantly reduced. Additionally, it can be seen from Figure 2 that ET-22, BL-99, and K56 only have one strip in the above swim lane, and no other small molecular bands are found, which also indicated that these three strains did not degrade gastrointestinal mucosal proteins.

### Platelet Aggregation Test

**FIGURE 3:**
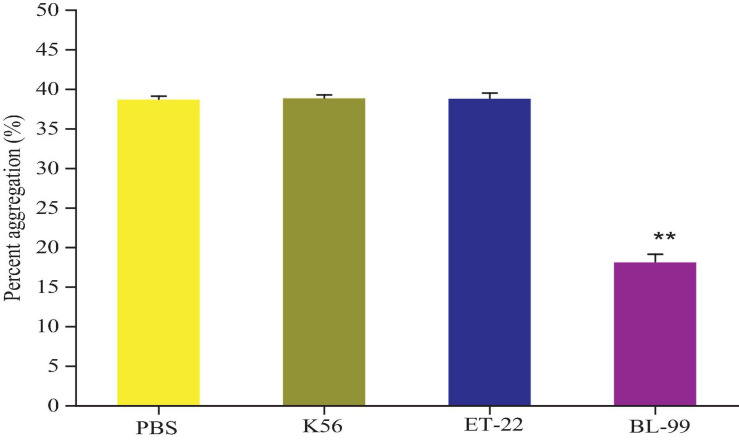
Detection of platelet aggregation ability in rabbit blood by tested bacterial strain samples. PBS was set as control group. Additionally, K56, ET-22, and BL-99 were marked as strains of *L. paracasei* K56 and ET-22 and *Bifidobacterium lactobacillus* BL-99, respectively. **p < 0.01.

According to the results of Figure 3, compared with the control group (PBS group), strains K56, ET-22, and BL-99 did not promote platelet aggregation, and BL-99 had a slight inhibitory ability to platelet aggregation. The experiment proved that the three strains did not cause platelet aggregation.

### Bacterial Reversion Mutation

As we can see from Table 1, under the test for *Salmonella* Typhimurium TA97, TA98, TA100, and TA102 strains in five dose groups 5,000, 1,000, 200, 40, and 80 μg/melamine with and without S9 system, the mutant colony counts in K56, ET-22, and BL-99 groups were not two times contrasted to the control. However, all the positive comparisons were sensitive. The results indicated that the Ames test for the tested strains, namely, K56, ET-22, and BL-99, were negative.

**TABLE 1:** Bacterial reverse mutation test conducted with K56, ET-22, and BL-99.

Tested group	Concentrations (μg/plate)	*Salmonella* Typhimurium TA97		*Salmonella* Typhimurium TA98		*Salmonella* Typhimurium TA100		*Salmonella* Typhimurium TA102	
		+S9	−S9	+S9	−S9	+S9	−S9	+S9	−S9
K56	5,000	118 ± 6	151 ± 7	56 ± 8	77 ± 4	168 ± 9	212 ± 6	339 ± 5	375 ± 6
	1,000	109 ± 6.	145 ± 4	51 ± 6	73 ± 5	172 ± 6	194 ± 4	325 ± 12	379 ± 5
	200	112 ± 6	154 ± 3	41 ± 6	86 ± 5	159 ± 6	215 ± 5	333 ± 11	370 ± 7
	40	126 ± 6	143 ± 4	48 ± 6	77 ± 5	172 ± 6	205 ± 10	336 ± 7	375 ± 9
	8	119 ± 6	132 ± 2	40 ± 6	76 ± 9	156 ± 4	203 ± 14	348 ± 12	368 ± 5
ET-22	5,000	119 ± 10	140 ± 7	50 ± 4	75 ± 4	161 ± 7	216 ± 8	333 ± 4	359 ± 7
	1,000	115 ± 10	143 ± 3	56 ± 3	83 ± 7	165 ± 11	200 ± 7	333 ± 7	362 ± 8
	200	130 ± 4	144 ± 7	46 ± 4	66 ± 6	167 ± 3	199 ± 10	324 ± 6	380 ± 4
	40	109 ± 14	145 ± 9	45 ± 3	70 ± 6	160 ± 5	191 ± 6	339 ± 9	370 ± 7
	8	124 ± 5	140 ± 6	44 ± 6	79 ± 7	163 ± 7	203 ± 5	333 ± 6	360 ± 8
BL-99	5,000	123 ± 8	131 ± 6	43 ± 3	69 ± 7	174 ± 4	209 ± 11	326 ± 6	372 ± 8
	1,000	116 ± 2	149 ± 9	48 ± 6	82 ± 7	162 ± 7	219 ± 11	334 ± 9	382 ± 5
	200	111 ± 5	143 ± 10	38 ± 5	74 ± 6	165 ± 11	222 ± 7	3,345 ± 6	360 ± 7
	40	107 ± 6	150 ± 7	37 ± 4	77 ± 1	154 ± 2	212 ± 12	343 ± 15	377 ± 5
	8	111 ± 9	152 ± 8	45 ± 5	77 ± 5	168 ± 6	191 ± 6	334 ± 9	362 ± 7
Control (water)	–	111 ± 4	154 ± 9	47 ± 3	85 ± 3	167 ± 5	205 ± 12	335 ± 7	372 ± 5
Control (DMSO)	–	110 ± 8	143 ± 8	42 ± 4	83 ± 4	163 ± 8	210 ± 11	331 ± 9	360 ± 6
2AA F	10	1,667 ± 170		4,827 ± 161		3,050 ± 163			
DHAQ	25							937 ± 50	
NaN_3_	2						2,747 ± 93		
Dexon	25		2,517 ± 217		1,183 ± 53				837 ± 82

*-S9, without metabolic activation; +S9, with metabolic activation; NaN_3_, sodium azide; Dexon, Dixone; 2AAF, 2-aminofluorene; DHAQ, 1,8-dihydroxyanthraquinone; DMSO, dimethyl sulfoxide.*

*The values (mean ± standard deviation) in the table show the CFUs.*

### Antibiotic Susceptibility

Herein, both antibiotic disc diffusion method and MIC measurement were applied to detect antibiotic susceptibility of the tested three strains. Tables 2, 3 show the sensitivity results of the tested strains ET-22, BL-99, and K56 to eight antibiotics. All of them were sensitive to ampicillin, gentamicin, streptomycin, erythromycin, penicillin G, tetracycline, and chloramphenicol.

**TABLE 2:** Results of antibiotic susceptibility testing by disc diffusion method.

Antibiotics	Sensitivity type		
	K56	ET-22	BL-99
Ampicillin (10 μg/disc)	S	S	S
Vancomycin (30 μg/disc)	n.r.	n.r.	S
Gentamicin (10 μg/disc)	S	S	S
Streptomycin (10 μg/disc)	S	S	S
Erythromycin (15 μg/disc)	S	S	S
Penicillin G (10 μg/disc)	S	S	S
Tetracycline (30 μg/disc)	S	S	S
Chloramphenicol (30 μg/disc)	S	S	S

*R, resistant; S, sensitive; n.r., not required. 15–18 mm; S ≥ 19 mm. Penicillin G: R ≤ 14 mm; I: -; S ≥ 15 mm. Ampicillin: R ≤ 16 mm; I: -; S ≥ 17 mm. Chloramphenicol: R ≤ 12 mm; I: 13–17 mm; S ≥ 18 mm. Gentamicin: R ≤ 12 mm; I: -; S ≥ 13 mm. Erythromycin: R ≤ 13 mm; I: 14–22 mm; S ≥ 23 mm. Vancomycin: R ≤ 14 mm; I: -; S ≥ 15 mm.*

**TABLE 3:** Antimicrobial susceptibility (MIC values) of *Bifidobacterium lactis* BL-99 and *Lacticaseibacillus paracasei* K56 and ET-22.

	EFSA cut-off (mg/L)		K56	ET-22	BL-99
	*Bifidobacterium*	*Lactobacillus* obligate heterofermentative			
Ampicillin	2	**4**	1	0.5	0.25
Erythromycin	1	1	0.0078	0.0156	0.0625
Vancomycin	2	n.r.	1024	1024	1
Chloramphenicol	4	4	4	4	2
Tetracycline	8	8	0.5	0.5	0.5
Gentamicin	64	16	0.5	0.5	32
Streptomycin	128	64	4	2	16
Penicillin G			0.0078	0.0063	0.03125

*n.r., not required.*

### Acute Oral Toxicity Study (14 Days Repeated Dose)

**FIGURE 4:**
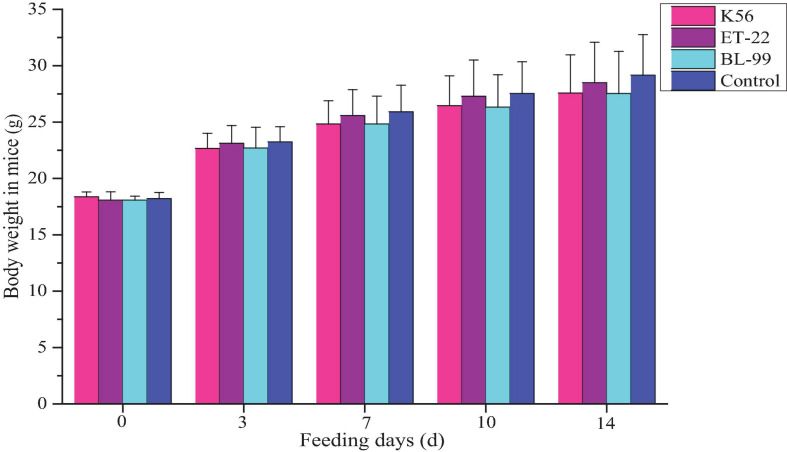
The dynamic change of body weight in ICR mice fed with BL-99, ET-22, and K56 strains within 14 days.

In this study, ICR mice were fed with different tested strains by gavage. During feeding for 14 days, all mice were in good action and mental state, without poisoning symptoms or even death. Furthermore, mice were dissected after 14-day intragastric administration. No pathogenic changes in viscera were observed *via* gross and microscopic examinations. Figure 4 shows the average body weight of mice in each group. Compared with the control, there was no significant difference in the above strains (*p* < 0.05).

### Subacute Oral Toxicity Study (28 Days Repeated Oral Dose)

**FIGURE 5:**
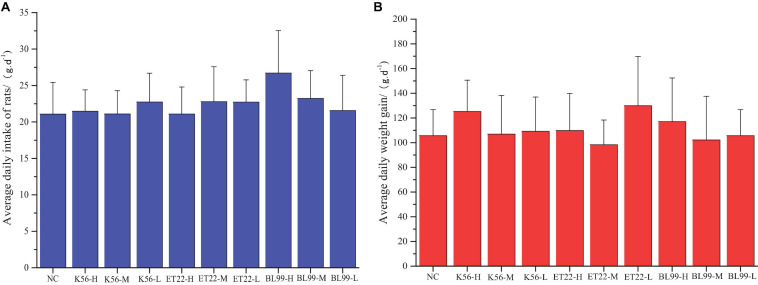
The effect of tested bacterial strains on the food intake and body weight increment of subacute rats. NC, PBS group; K56-H, ET22-H, and BL99-H denoted fed with high dose of K56, ET-22, and BL-99 for rats. K56-M, ET22-M, and BL99-M denoted fed with medium dosed (half of high dose) of K56, ET-22, BL-99 for rats, respectively. K56-L, ET22-L, and BL99-L denoted fed with medium dose (quarter of high dose) of K56, ET-22, and BL-99 for rats, respectively. The highest dose of K56-L, ET22-L, and BL99-L was 5.25 × 10^11^, 5.25 × 10^11^, and 7.5 × 10^11^ CFU/kg, respectively. **(A)** Average daily intake of rats. **(B)** Average daily weight gain.

Comparison of weights in experimental animals between the control and the tested material has long been accepted as a sensitive indicator of chemically induced changes in organs or toxicological effects. In this study, after the rats were given different tested strains by gavage, the rats in each group were in good growth, behavior, and mental state, and no poisoning symptoms or death were observed. The figure above shows the feed intake (Figure 5A) and body weight (Figure 5B) of the rats in each experimental group increased. The body weight increase of the tested bacterial strain group was not significantly different from that of the control group (*p* > 0.05).

### Changes of Blood Biochemical Indexes and Physiological Indexes

According to the results in Table 4, when the gavage doses of both ET-22 and K56 were 2.62 × 10^11^ and 1.31 × 10^11^ CFU/kg, respectively, in addition, BL-99 was at the dose of 1.88 × 10^11^ CFU/kg, there was no significant difference in the above biochemical indexes compared with the control group (*p* > 0.05). Therefore, blood biochemical indices of medium (M) and low doses (L) of K56, ET-22, and BL-99 were not significantly different from those of the control group, and the analysis data concluded that blood biochemical indexes were safe under this dose.

**TABLE 4:** Changes of blood biochemical indexes.

Index	Unit	NC	K56-H	K56-M	K56-L	ET22-H	ET22-M	ET22-L	BL99-H	BL99-M	BL99-L
ALT	U/L	36.44 ± 4.37	28.83 ± 9.14**	40.77 ± 11.29	41.07 ± 11.11	27.84 ± 9.35	40.55 ± 13.18	32.85 ± 10.16	24.4 ± 8.14**	28.26 ± 7**	30.99 ± 11.32
AST	U/L	90.67 ± 12.46	87.38 ± 27.57	102.08 ± 28.94	104.4 ± 32.4	91.91 ± 30.71	88.33 ± 28.27	74.6 ± 20.87	95.33 ± 37.57	55.05 ± 19.8**	79.67 ± 7.13
GGT	μmol/L	6.51 ± 1.56	6.35 ± 1.81	6.81 ± 0.99	6.06 ± 1.2	6.57 ± 1.61	7. ± 2.7	7.18 ± 1.41	6.07 ± 1.37	7.78 ± 1.25**	7.6 ± 2.64
ALP	U/L	148.51 ± 30.77	84.24 ± 34.84**	176.14 ± 65.87	184.8 ± 73.82	96.86 ± 27.77**	175.1 ± 64.53	129.06 ± 50.13	84.57 ± 26.66**	107.95 ± 48.93*	116.58 ± 34.77
BUN	mmol/L	5.32 ± 2.16	4.38 ± 2.25	6.13 ± 2.11	6.46 ± 2.79	5.67 ± 2.6	6.29 ± 1.82	5.05 ± 2.05	4.65 ± 1.81	4.67 ± 2.64	4.73 ± 2.22
CR	μmol/L	34.37 ± 9.14	26.5 ± 11.3	29.57 ± 6.9	33.28 ± 9.64	34.00 ± 8.90	31.75 ± 8.72	27.25 ± 9.36	32.9 ± 10.1	24.7 ± 9.07**	27.63 ± 10.24
GLU	mmol/L	5.48 ± 1.57	4.12 ± 0.28	5.97 ± 2.43	6.63 ± 2.6	4.43 ± 1.77	5.95 ± 0.88	5.29 ± 1.71	3.72 ± 1.64**	6.54 ± 2.37	6.13 ± 2.17
TP	g/L	35.73 ± 14.6	26.8 ± 13.31	32.2 ± 9.66	39.13 ± 11.25	32.51 ± 14.13	35.28 ± 11.63	21.02 ± 10.72	27.34 ± 12.4	25.89 ± 11.92*	29.99 ± 14.56
ALB	g/L	19.09 ± 6.46	14.87 ± 6.04	18.84 ± 4.84	20.72 ± 6.22	17.19 ± 6.21	18.84 ± 5.05	16.41 ± 5.3	15.87 ± 5.06	14.72 ± 5.56*	15.03 ± 5.04
TG	mM	0.27 ± 0.09	0.3 ± 0.09**	0.3 ± 0.09	0.3 ± 0.08	0.31 ± 0.12	0.28 ± 0.07	0.28 ± 0.13	0.31 ± 0.14	0.28 ± 0.08	0.26 ± 0.08
TC	mmol/L	1.2 ± 0.49	1.2 ± 0.38	1.2 ± 0.38	1.23 ± 0.5	1.06 ± 0.48	1.18 ± 0.42	0.91 ± 0.41	0.95 ± 0.45	1.01 ± 0.44	0.86 ± 0.38
K	mmol/L	4.56 ± 0.56	4.94 ± 0.69	4.4 ± 0.55	4.49 ± 0.99	4.55 ± 0.75	4.48 ± 0.95	4.76 ± 1.05	4.25 ± 0.49	4.26 ± 0.93	4.36 ± 1
Na	mmol/L	136.37 ± 8.5	129.04 ± 12.2	128.84 ± 9.34	131.03 ± 27.02	121.97 ± 6.58**	132.53 ± 25.21	134.45 ± 30.07	125.98 ± 0.4*	131.15 ± 26.01	133.63 ± 26.91
Cl	mmol/L	102.63 ± 6.1	99.27 ± 9.14	93.12 ± 9.4	93.77 ± 19.71	90.71 ± 5.47**	96.74 ± 19.37	91.42 ± 17.9	94.93 ± 7.7	101.25 ± 23.84	98.14 ± 20.52
PT	s	10.77 ± 0.37	10.07 ± 0.72	10.66 ± 2.25	10.36 ± 0.99	10.15 ± 0.45	10.6 ± 0.94	10.49 ± 0.7	10.02 ± 0.44*	10.59 ± 1.31	10.58 ± 0.85
PT-INR	s	0.85 ± 0.03	0.8 ± 0.06	0.82 ± 0.11	0.86 ± 0.09	0.81 ± 0.04	0.85 ± 0.08	0.83 ± 0.06	0.79 ± 0.04*	0.86 ± 0.13	0.86 ± 0.1
APTT	s	17.3 ± 1.28	16.94 ± 2.42	15.12 ± 4.73	16.28 ± 2.06	17.01 ± 1.13	16.11 ± 2.19	15.57 ± 4.22	16.97 ± 0.93	16.63 ± 2.09	16.95 ± 2.07

**p < 0.01; *p < 0.05; n = 20.

*ALT, alanine transaminase; AST, aspartate aminotransferase; GGT, gamma-glutamyltransferase; ALP, alkaline phosphatase; BUN, blood urea nitrogen; CR, serum creatinine; GLU, glucose; TP, total protein; ALB, albumin; TG, triglyceride; TC, total cholesterol; K, serum potassium; Na, serum natrium; Cl, chlo rine; PT, prothrombin time; PT-INR, International standard value of prothrombin time; APTT, activated partial thromboplastin time.*

As can be seen from Table 5, a series of hematological parameter analysis results showed that when ET-22 was given an intragastric dose of 2.62 × 10^11^ CFU/kg, K56 was given an intragastric dose of 2.62 × 10^11^ CFU/kg, and BL-99 was given an intragastric dose of 1.88 × 10^11^ CFU/kg; there was no significant difference in blood parameters measured between the treatment group and the sterile water control group (*p* > 0.05). Furthermore, across all measurements, there was an insignificant reduction or abnormality in blood indicators of anemia (such as RBC, HGB, HCT, MCV, MCH, NEU, and MCHC) between the tested group and the control. In addition, blood parameters (such as lymphocytes, monocytes, eosinophils, neutrophil, and basophils) that are markers of blood infection in all test groups were comparable with those in the PBS control group, and no significant changes were observed, indicating no infection. Blood biochemical parameters were similar between the treatment group and the control and did not show any treatment-related infection or anemia toxicity.

**TABLE 5:** Changes of blood physiological indexes.

	NC	K56-H	K56-M	K56-L	ET22-H	ET22-M	ET22-L	BL99-H	BL99-M	BL99-L
WBC	6.25 ± 3.75	5.90 ± 1.55	6.59 ± 2.68	6.84 ± 2.14	6.08 ± 2.79	7.5 ± 3.93	6.58 ± 3.53	5.9 ± 2.24	8.2 ± 2.12	8.42 ± 3.16
RBC	6.32 ± 1.9	6.67 ± 0.31*	6.02 ± 1.82	6.7 ± 0.34	6.2 ± 1.84	6.68 ± 0.68	6.11 ± 1.71	6.75 ± 0.19	6 ± 0.3**	6.64 ± 0.56
HGB	134.3 ± 11.54	144.14 ± 8.17**	122.5 ± 36.81	132.18 ± 10.52	131.27 ± 38.81	127.7 ± 19.19	120.93 ± 33.17	144.41 ± 3.78**	138 ± 7.5	138.05 ± 14.57
MCV	45.15 ± 13.5	52.35 ± 2.11**	46.71 ± 14.18	50.05 ± 1.9	47.16 ± 14.06	50.19 ± 1.79	46.38 ± 12.29	51.34 ± 2.05**	50.4 ± 1.6	48.72 ± 2
PLT	687.76 ± 214.94	716.27 ± 113.6	659.88 ± 204.09	702.14 ± 86.13	714.9 ± 218.14	693.09 ± 124.33	655.92 ± 201.45	705.71 ± 58.20	700.95 ± 104.06	704.45 ± 102.69
NEU%	22.81 ± 9.49	25.85 ± 5.79	25.90 ± 8.16	25.16 ± 4.4	23.05 ± 8.54	25.16 ± 7.34	21.81 ± 9.25	23.25 ± 3.27	23.5 ± 3.17	26.22 ± 9.58
LYM%	50.15 ± 15.77	55 ± 6.37	50.64 ± 14.97	57.11 ± 6.78	49.67 ± 16.16	53.53 ± 10.77	52.49 ± 19.91	55.38 ± 5.9	58.43 ± 3.66*	57.31 ± 10.22
EOS%	0.29 ± 0.15	0.35 ± 0.16	0.47 ± 0.24	0.51 ± 0.2	0.44 ± 0.35	0.68 ± 0.52	0.4 ± 0.25	0.42 ± 0.26	0.55 ± 0.21	0.51 ± 0.25
BAS%	0.15 ± 0.1	0.09 ± 0.12	0.23 ± 0.35	0.5 ± 0.86	0.23 ± 0.3	0.23 ± 0.17	0.21 ± 0.13	0.34 ± 0.36*	0.21 ± 0.1	0.22 ± 0.15
NEU#	1.62 ± 1.19	1.49 ± 0.44	1.88 ± 0.75	1.73 ± 0.67	1.55 ± 0.86	2.66 ± 3.81	1.68 ± 1.6	1.35 ± 0.52	1.93 ± 0.62**	2.18 ± 1.25
LYM#	3.39 ± 1.94	3.27 ± 0.98	3.53 ± 1.4	3.91 ± 1.35	3.31 ± 1.52	3.87 ± 1.77	3.83 ± 2.02	3.32 ± 1.46	4.78 ± 1.26**	4.89 ± 2.36
PDW	9.09 ± 12.75	9.6 ± 1.7	8.41 ± 2.67	9.11 ± 1.26	9.46 ± 2.7	9.5 ± 2.14	8.7 ± 2.87	9.41 ± 1.44	9.28 ± 1.53**	9.85 ± 1.97
P-LCR	0.21 ± 0.16	0.19 ± 0.04	0.2 ± 0.14	0.18 ± 0.05	0.2 ± 0.11	0.19 ± 0.08	0.20 ± 0.09	0.19 ± 0.05	0.18 ± 0.04**	0.19 ± 0.06
P-LCC	131.73 ± 47.47	135.72 ± 37.57*	98.5 ± 28.27	104.12 ± 25.3	130.14 ± 46.37	99.05 ± 33.86	97.5 ± 43.2	129.04 ± 41.58	98.41 ± 30.27*	105.77 ± 33.4
MON%	18.60 ± 6.4	18.71 ± 2.58	16.36 ± 5.16	16.54 ± 3.78	18.67 ± 6.65	16.15 ± 3.79	15.46 ± 4.43	20.61 ± 4.43	16.97 ± 2.37	16.05 ± 2. 5
MON#	1.26 ± 0.79	1.11 ± 0.37	1.18 ± 0.55	1.11 ± 0.38	1.22 ± 0.54	1.46 ± 1.73	1.08 ± 0.47	1.19 ± 0.45	1.4 ± 0.4**	1.26 ± 0.4
EOS#	0.06 ± 0.16	0.02 ± 0.01	0.04 ± 0.02	0.04 ± 0.02	0.06 ± 0.12	0.05 ± 0.06	0.04 ± 0.04	0.02 ± 0.01	0.04 ± 0.02	0.04 ± 0.02
BAS#	0.008 ± 0.16	0.01 ± 0.01	0.02 ± 0.04	0.03 ± 0.06	0.036 ± 0.09	0.04 ± 0.04	0.02 ± 0.021	0.02 ± 0.02*	0.01 ± 0.01	0.01 ± 0.01
HCT	31.14 ± 9.31	34.94 ± 2.22	30. ± 9	33.2 ± 1.94	31.97 ± 0.44	33.02 ± 2.12	30.23 ± 8.32	34.6 ± 0.13	32.7 ± 1.71**	32.98 ± 2.13
MCH	19.6 ± 5.72	21.59 ± 0.58	19.03 ± 5.76	20.8 ± 1.22	19.37 ± 5.75	20.84 ± 1.02	19.15 ± 5.15	21.4 ± 0.68	21.31 ± 0.65	20.83 ± 1.04
MCH C	420.14 ± 87.85	412.91 ± 14.4	373.06 ± 114.01	414.55 ± 11.74	376.23 ± 112.57	415.18 ± 14.65	380.74 ± 102.14	417.23 ± 11.18	423.64 ± 7.74	426.18 ± 10.83
RDW-CV	8.13 ± 2.4	8.56 ± 0.56	7.69 ± 2.31	8.55 ± 0.46	8.28 ± 2.45	8.53 ± 0.6	7.92 ± 2.19	8.7 ± 0.44	8.55 ± 0.52	8.54 ± 0.6
RDW-SD	24.1 ± 7.17	27.1 ± 0.89*	24.54 ± 7.39	26.37 ± 0.54	24.86 ± 7.46	26.44 ± 0.33	24.33 ± 6.38	26.75 ± 0.76	26.35 ± 0.37	26.09 ± 0.43
MPV	8.85 ± 2.51	9.66 ± 1.17	8.88 ± 7.39	9.84 ± 1.14	8.59 ± 2.4	9.87 ± 1.48	9 ± 2.48	9.61 ± 1.6	9.6 ± 0.9	9.76 ± 0.97
PCT	0.5 ± 0.05	0.69 ± 0.14**	0.47 ± 0.16	0.49 ± 0.14	0.68 ± 0.17**	0.56 ± 0.24	0.49 ± 0.2	0.67 ± 0.12**	0.55 ± 0.11	0.56 ± 0.169

**p < 0.01; *p < 0.05; n = 20.

To sum up, the concentration of K56, ET-22, and BL-99 (2.62 × 10^11^, 2.62 × 10^11^, 1.88 × 10^11^ CFUs/kg BW of mice, respectively) considered the no-observed-adverse-effectlevel (NOAEL) in this study. Our findings were consistent with other equivalent oral repeated toxicity studies of lactic acid bacteria, which have shown that dosing levels ranging from 10^8^ to 10^10^ CFU/day have no adverse or toxicological effects on experimental rats according to acute or subacute toxicity studies (Jia et al., 2011; Owaga et al., 2014; Han et al., 2021).

### Histopathological Examination and Bacterial Translocation

**FIGURE 6:**
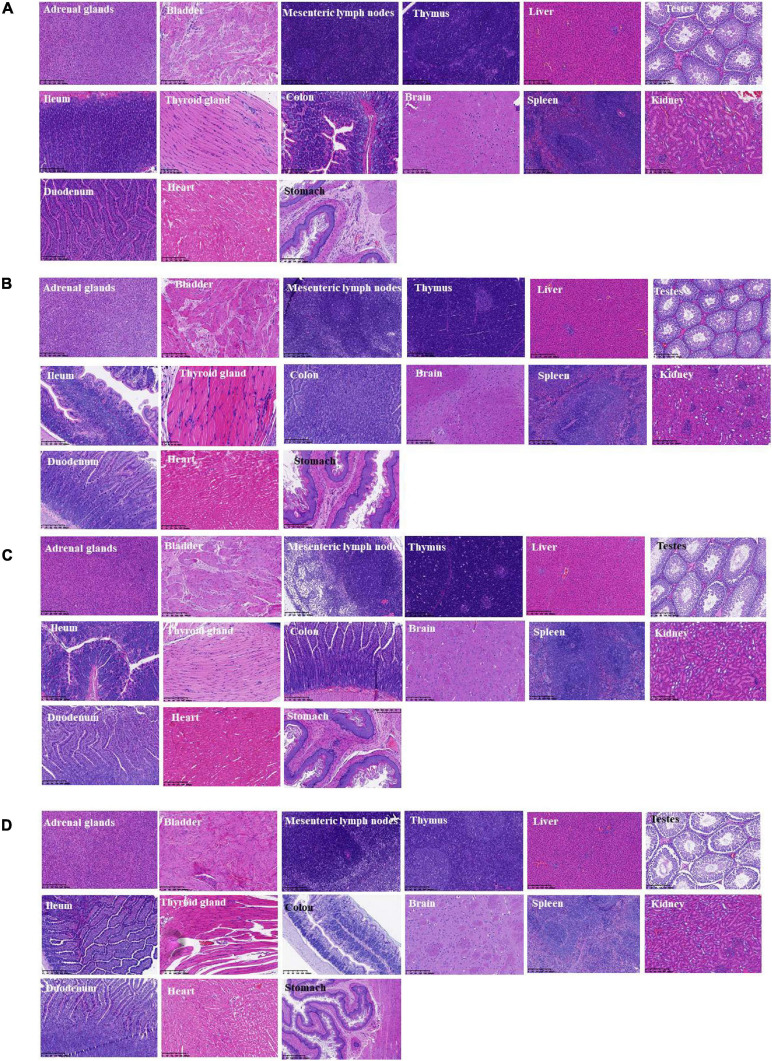
Histopathological detection of feeding rats on 28-day acute oral toxicity study. **(A)** BL-99 group; **(B)** K56 group; **(C)** ET-22 group; **(D)** PBS group.

During the whole observation period of this experiment, the rats in each group were in good condition, with no color changes in appearance, fur, and mucosa, barrier-free movement, and no abnormalities in eating and breathing. All SD rats were killed and dissected on the 28th day. Visual observation showed that the appearance of all organs in the rats was complete and good, with normal color. No obvious bleeding or edema was observed. Additionally, HE was carried out on the part of the viscera staining slice observation. Compared with the control group (shown in Figure 6D), main organs in rats did not appear abnormal without significant differences by microscopic observation. Among them, glomerular, renal tubular structure was normal. Capillary congestion does not appear to be expanded. No inflammatory cells were infiltrated (Figures 6A–D). Moreover, the size and shape of hepatocytes and hepatic lobules were normal, without infiltration of lymphocytes and macrophages. No abnormal changes were observed in the heart, clear myocardial cells, regular arrangement of myocardial fibers, and no inflammatory cell infiltration. The structure and morphology of the spleen were normal without lymphocyte swelling. Thymus tissue skin and medulla were clearly demarcated, thymus structure was intact, and no obvious abnormalities were observed. Additionally, according to the results of bacterial translocation test (Table 6), after ingestion of the three tested bacterial strains (two strains of *L. paracasei* K56 and ET-22 and *B. lactis* BL-99), these three strains would not appear in the heart, liver, spleen, lung, kidney, and brain, indicating that consuming *L. paracasei* K56 and ET-22 and *B. lactis* BL-99 as probiotics had no translocation ability to these organs.

**TABLE 6:** The incidence of bacterial translocation in rate from the control and treated groups.

Tissue/organ	ET-22 (5.25 × 10^11^ CFU/kg)		K56 (5.25 × 10^11^ CFU/kg)		BL-99 (7.5 × 10^11^ CFU/kg)		PBS (control)	
	BHI	MRS	BHI	MRS	BHI	MRS	BHI	MRS
Heart	0/10	0/10	0/10	0/10	0/10	0/10	0/10	0/10
Liver	0/10	0/10	0/10	0/10	0/10	0/10	0/10	0/10
Kidne y	0/10	0/10	0/10	0/10	0/10	0/10	0/10	0/10
Spleen	0/10	0/10	0/10	0/10	0/10	0/10	0/10	0/10
Lung	0/10	0/10	0/10	0/10	0/10	0/10	0/10	0/10

*Values are presented as the number of animals that were positive for bacterial translocation/total number of animals tested per group. Numbers in brackets represents the number of colonies counted (CFU/g of tissue). MRS, De Man Rogosa and Sharpe agar; BHI, brain heart infusion agar.*

## Discussion

Human host under normal health conditions is protected by layers of effective constitutive and specific defense mechanisms. The mucosal surface of the gastrointestinal stress (GIT) is covered by a thick mucus layer that helps to control the normal microbiota of the intestinal tract and contain it at their natural resident sites and prevent them from invading and entering the bloodstream (Frenkel and Ribbeck, 2015b). Hence, mucin forms a protective layer on the epithelial surface of the entire gastrointestinal mucosa and plays a key role in host defense (Frenkel and Ribbeck, 2015a). Mucus decomposition was reported to be one of the pathogenicity of many high-risk pathogens, such as *Vibrio cholerae*, *Helicobacter pylori*, *Salmonella*, and *Shigella* (Zhou et al., 2001). These all indicated that the three strains would not grow, with gastrointestinal mucosal protein as carbon source. Therefore, it is necessary to evaluate the decomposition activity of the new strain on gastrointestinal mucosa proteins. Basal media (glucose-free MRS broth medium, 0.3% mucin basal medium, 1.0% glucose basal medium, and 1.0% glucose 0.3% mucin basal medium) (Kang et al., 2019) were specially used for mucin degradation test. Our results demonstrated that the three strains were found to be negative for mucin degradation, in agreement with those of earlier studies (Pradhan et al., 2019). In addition, earlier studies reported no myxolysis activity in potential probiotic strains (Boyiri and Onyango, 2015).

Opportunistic translocation of microorganisms into the bloodstream may also induce disease through platelet aggregation, contributing to the pathogenesis of infective endocarditis. Platelet aggregation induced by *Lactobacillus* was considered to be an important contributor to the occurrence and development of *Lactobacillus* endocarditis (IE) (Zhou et al., 2005; Encarnacion et al., 2016). Platelet aggregation is the initial step in thrombogenesis and IE, so the platelet-aggregating strains are more pathogenic than the non-aggregating strains. Previous reports have suggested that some species of *Lactobacillus* are aggregative (Harty et al., 1993). In addition, it has also been reported that lactic acid bacteria cannot induce platelet aggregation (Pradhan et al., 2019), indicating that aggregation characteristics are highly strain specific. Therefore, the aggregation characteristics of each strain must be examined. The experiment proved that the three strains did not cause platelet aggregation. Interestingly, BL-99 slightly inhibited platelet aggregation. Recently, *L. casei* CRL 431 was found to improve endothelial and platelet functionality in pneumococcal infection models (Haro and Medina, 2019), which means that BL-99 strain detected in this study may have medical development and application potential.

It is well established that long-term toxicity of chemicals could be caused by their ability to generate changes in the DNA sequence through the process of mutagenesis (Szyf, 2011). The investigation whether probiotics have gene progenicity is also very important to evaluate the safety of probiotics. The *Salmonella* reversion-based *Salmonella*/microsome test (Ames test) developed in 1973 by Bruce Ames (Ames et al., 1973) is a widely used bioassay to determine mutagenic potential of compounds in toxicological risk assessment (Zwart et al., 2018). Thus, Ames is the most widely used method for mutagenicity testing in probiotics, including *L. plantarum*, *L. paracasei*, *B. adolescentis*, and *P. acidilactici* (Chiu et al., 2013; Tseng et al., 2015; Lin et al., 2018; Liao et al., 2019). Herein, our findings indicated that the tested strain, namely, K56, ET-22, and BL-99, were negative in Ames test, which indicated that the tested strains had no genetic mutagenicity.

The determination of antibiotic sensitivity of microorganisms is of great significance to evaluate the safety of bacterial strains (Das et al., 2020). Only non-resistant strains are safe to use in humans. Therefore, the three potentially probiotic strains were subjected to antibiotic susceptibility testing using the agar diffusion method and MIC measurement. According to the CLSI drug sensitivity test standard (CLSI, 2015), the antibiotic sensitivity of the strains were judged. Generally, the more sensitive the strain was to antibiotics, the weaker the activity around the drug-sensitive paper was, and the larger the diameter of the transparent circle, namely, the antibacterial circle, was finally formed. It has been reported that some type of *lactobacilli* species found a high level of resistance to aminoglycosides and ciprofloxacin (Tejinder et al., 2012). Moreover, some *lactobacilli* had previously been described to be instrinsically resistant to antibiotics. In most cases, the resistance is naturally occurring, non-transferrable, and has been attributed as a species and/or genus characteristic (Umar et al., 2020). Nonetheless, it has been reported that multiple drug resistance was observed for some of the tested strains, whereas no conjugal transfer of the antibiotic gene markers was observed (Guo et al., 2017). In the previous study, the antibiotic susceptibility of 33 *Lactobacillus* strains isolated from traditional dairy products was evaluated (Guo et al., 2017). The results showed that all strains were sensitive to gentamicin and erythromycin, which was in agreement with this study. Additionally, recent research also showed that the *Lactobacillus* species isolated from Iranian Jug Cheese were susceptible or intermediate susceptibility to polyketides (tetracycline), β-lactams (penicillin, ampicillin), amphenicols (chloramph-enicol), and macrolides (erythromycin) (Mahmoudi et al., 2020).

Though *in vitro* evaluations of virulence traits were a prerequisite of a probiotic candidate strain, *in vivo* studies in appropriate animal model were essential for confirming their safety. The oral toxicity studies were considered a standard for establishing safety of a bacterial strain (Khalkhali and Mojgani, 2018). After given gavage for 14 and 28 days, mice with different tested strains were in good health, with no acute and subacute toxicity b examined. Additionally, referring to pathological and physiological states of animals and humans, the most sensitive target for toxic substances can be measured through the hematopoietic system. Biochemical assays can be used for detecting moderate to mild deficiency of nutrients or an imbalance in nutrient metabolism (Swendseid, 1987). Moreover, according to biochemical indexes, the three tested strains are safe and non-toxic at this gavage dose. Consistent with Soodabeh results (Khalkhali and Mojgani, 2018), we also observed the decreased serum levels of ALT and ALP in the treated animals, which might suggest a better functioning of the liver in the treated animals than the controls. It has been well acknowledged that increased peripheral blood neutrophils or eosinophils are well-known indicators of bacterial infection (Dahl et al., 1976; Zhou et al., 2000). Therefore, detecting blood physiological indexes affected by strains is of great importance. Our findings showed that intake K56, ET-22, and BL-99 with concentration of 10^11^ CFUs/kg BW has no observed adverse effect in blood physiological indexes of mice. Previous research has also reported that the no-observed-adverse-effect level of *Bacillus licheniformis* was found to be greater than 1.1 × 10^11^ CFU/kg BW, neither significant differences in serum biochemical and hematological analyses nor histopathological changes in organs or tissues were found when compared with the control groups (Nithya et al., 2012). Importantly, histopathological examination showed that no inflammatory cells were infiltrated in mice organs after intake of the tested strains, indicating that the tested strains, namely, K56, ET-22, and BL-99 have no adverse effect. A translocation-positive animal was defined as an animal that had at least one tissue sample (including blood) containing one or more viable bacterial cells (Zhou et al., 2000). Translocation of probiotics can lead to infection, mainly bacteremia, septicemia, or endocarditis (Min-Tze, 2010). Therefore, it is very important to evaluate the translocation ability of probiotics. Our results showed that the tested three strains did not contribute to bacterial translocation *in vivo.* Similarly, Pradhan et al. (2019) found that MTCC5690 and MTCC5689 not detected in the tissues and organs under test with random state expansion increased DNA (RAPD) fingerprinting method by arbitrary selection of primers to identify tissue sample translocation microbes. Abe et al., 2010) also reported that there were no disturbance of epithelial cells and mucosal layer in the ileum, cecum, and colon. Moreover, for comprehensively evaluating the safety of potential probiotics, clinical trials need to be further carried out in the future, so as to systematically evaluate the safety of the tested strains.

## Conclusion

Herein, a comprehensive assessment regarding safety and toxicity using *in vitro* and *in vivo* approaches was carried out for *L. paracasei* K56 and ET-22 and *B. lactis* BL-99. These strains were found to be negative for mucin degradation, platelet aggregation test, and antibiotic susceptibility, along with no genetic mutagenicity in Ames assay. Additionally, no evidence of pathogenicity and mortality during the oral toxicity study has been obtained. Overall, this study suggests that K56, ET-22, and BL-99 are non-pathogenic and likely to be safe for incorporation in food formulations, contributing to screening for safe potential probiotic bacterial strains that might be used for producing functional foods.

## Data Availability Statement

The original contributions presented in the study are included in the article/ **Supplementary Material**, further inquiries can be directed to the corresponding author/s.

## Ethics Statement

The animal study was reviewed and approved by SYXK (Zhejiang) 2014-0008.

## Author Contributions

QC and W-LH designed the research. HYL, WZ, HHL, and TW carried out the research activities. HYL analyzed the data and wrote the manuscript. TS supervised the research and data. QC and W-HL revised and submitted the final version of the manuscript. All authors have read and agreed to the published version of the manuscript. Authorship is limited to those who have contributed substantially to the work reported.

## Conflict of Interest

WZ, W-HL, TS, and W-LH were employed by the company Inner Mongolia Dairy Technology Research Institute Co., Ltd. and Inner Mongolia Yili Industrial Group Co., Ltd. The remaining authors declare that the research was conducted in the absence of any commercial or financial relationships that could be construed as a potential conflict of interest.

## Publisher’s Note

All claims expressed in this article are solely those of the authors and do not necessarily represent those of their affiliated organizations, or those of the publisher, the editors and the reviewers. Any product that may be evaluated in this article, or claim that may be made by its manufacturer, is not guaranteed or endorsed by the publisher.
